# Anatomical adaptation of a custom 3D prosthesis for canine laryngeal stenosis: a preliminary cadaveric study

**DOI:** 10.4142/jvs.25272

**Published:** 2026-06-18

**Authors:** Sandra Lopez-Minguez, Carolina Serrano-Casorran, Ignacio Colom-Diaz, Cantal del Rio-Martinez, Manuel Alaman, Cristina Gonzalez-Pastor, Julia Laliena, Cristina Bonastre, José B Rodriguez

**Affiliations:** 1 Department of Animal Pathology, Veterinary School, University of Zaragoza, Zaragoza 50013, Spain.; 2 Minimally Invasive Research Group (GITMI), University of Zaragoza, Zaragoza 50013, Spain.; 3 Department of Mechanical Engineering, School of Engineering and Architecture, University of Zaragoza, Zaragoza 50018, Spain.

**Keywords:** Vocal cord paralysis, brachycephalic airway syndrome, airway stenting, veterinary prosthesis, dogs

## Abstract

**Importance:**

Laryngeal stenosis is a frequent and life-threatening cause of upper airway obstruction in dogs. Current surgical options, although effective, are invasive and associated with high morbidity. Species-specific laryngeal stents may provide a safer alternative.

**Objective:**

To evaluate the anatomical adaptation and feasibility of a novel custom three-dimensional (3D)-printed laryngeal prosthesis designed for canine laryngeal stenosis in a cadaveric model.

**Methods:**

A prototype stent was produced using 3D printing and assessed in eight canine cadavers of different breeds and skull conformations. Glottic opening was measured at baseline, after endotracheal intubation, and following stent placement using calibrated laryngoscopic imaging. Anatomical fit, displacement, and epiglottic function were evaluated.

**Results:**

After stent placement, mean glottic opening was consistently smaller than that achieved with endotracheal tubes (e.g., size L: 17.3 ± 2.26 mm vs. 23.65 ± 6.72 mm), although this difference was not statistically significant, suggesting a more physiologic dilation. Optimal anatomical adaptation was observed in five of eight cases. Mild displacement occurred only in undersized implants, and epiglottic closure was preserved in all specimens.

**Conclusions and Relevance:**

This preliminary cadaveric study demonstrates that a custom canine-specific 3D laryngeal prosthesis can achieve stable anatomical integration without excessive glottic expansion or interference with epiglottic function. Although cadaveric testing cannot assess inflammatory responses, the limited dilation observed indicates low radial force. These findings support the potential of this device as a species-specific alternative to traditional surgical approaches. *In vivo* evaluation with medical-grade materials is warranted to confirm safety, tolerability, and long-term efficacy.

## INTRODUCTION

Disorders of the larynx are a frequent source of upper-airway obstruction in dogs [1]. Clinical presentation of laryngeal stenosis varies from subtle exercise intolerance to acute, life-threatening distress [1,2,3]. Effective management is critical to improve quality of life and survival [1,4]. Despite diagnostic and surgical advances, laryngeal disease remains complex and demands continued innovation and refinement [2,3,4].

Anatomically, the larynx is a sophisticated structure composed of nine cartilages that together with the intrinsic laryngeal muscles coordinate glottic opening and closure to regulate airflow and protect the lower airway from aspiration [5]. When these kinematics are disrupted, predictable patterns of disease arise [5,6].

Among the most frequently observed disorders is laryngeal paralysis, predominantly in older large- and giant-breed dogs [2,7,8]. Failure of arytenoid abduction during inspiration narrows the rima glottidis and increases resistance [8]. While congenital forms occur in specific breeds, the acquired form is far more common and is associated with polyneuropathy, trauma, or idiopathic causes; in many patients it heralds progressive, generalized neurologic disease. Clinical management typically involves surgery, most often unilateral arytenoid lateralization [3,8,9].

Another common disorder is laryngeal collapse [10]. Often seen in brachycephalic dogs and frequently coexisting with brachycephalic obstructive airway syndrome, results from progressive loss of cartilaginous support under chronic negative inspiratory pressure [10,11]. Severe cases may require cricoarytenoid–thyroarytenoid lateralization, partial laryngectomy, or permanent tracheostomy [4,11,12]. Although lifesaving, these procedures carry high complication rates, including aspiration pneumonia, stoma stenosis, and recurrent obstruction [12,13,14,15,16].

In contrast to advances in imaging and surgery, prosthetic devices for laryngeal disease have lagged [3,17]. Traditional laryngeal stents used in dogs are commonly adapted from human devices and often fail to provide an optimal fit due to anatomical differences, leading to inadequate support, excessive dilation, and secondary complications such as inflammation or mucosal injury [2,3]. In human airway applications, fenestrated silicone stents have reduced mucus retention, migration, and granulation compared with cylindrical designs, likely owing to improved airflow dynamics and localized tissue adaptation [18]. Veterinary case series describing the use of human-derived or silicone laryngeal stents in dogs have been reported, including preliminary clinical experiences, but none specifically addresses the functional demands of the canine larynx [2,7]. These limitations support the need for anatomically tailored, species-specific prostheses that minimize radial pressure and preserve mucosal function.

Three-dimensional (3D) printing offers a powerful approach for prototyping veterinary airway devices [7]. Custom 3D-printed models enable precise anatomical adaptation and iterative functional testing before clinical use. While 3D printing has been applied to other laryngeal interventions in dogs, such as arytenoid abductor devices evaluated *ex vivo* [7], the development of canine-specific laryngeal stents remains preliminary and underexplored.

In the development of airway prostheses intended to integrate with complex anatomical structures, identifying an optimal final design often requires multiple iterative modifications. 3D printing facilitates this process by enabling rapid, reproducible, and incremental design adjustments, allowing continuous refinement of geometry until adequate anatomical integration is achieved. This approach is particularly valuable in veterinary medicine, where marked interbreed anatomical variability demands devices that are fully adapted to species-specific and size-dependent anatomical features.

This study introduces a novel anatomical laryngeal stent prototyped with a 3D printer. The device is engineered to align with the canine laryngeal anatomy, and to exert minimal radial force on surrounding structures crucially. The stent expands only the ventral portion of the rima glottidis, avoiding dorsal over-dilation that could impair epiglottic function.

The aim of this preliminary cadaveric study was to evaluate anatomical integration and mechanical fit, focusing on positioning, glottic opening, and resistance to displacement. We hypothesized that targeted ventral expansion and contour-conforming geometry would provide secure placement with limited radial stress, supporting subsequent refinement and future *in-vivo* investigation.

## METHODS

### Prosthesis design

The design of the laryngeal stent was guided from the outset by a set of predefined anatomical and functional objectives. These included: preservation of complete laryngeal closure by the epiglottis during swallowing; sufficient flexibility to allow anatomical and physiological adaptation to the canine larynx; secure fixation with resistance to migration; maximization of the free laryngeal lumen to avoid unnecessary airflow obstruction; and promotion of effective mucociliary clearance by avoiding circumferential coverage and excessive material bulk.

**Fig. 1:**
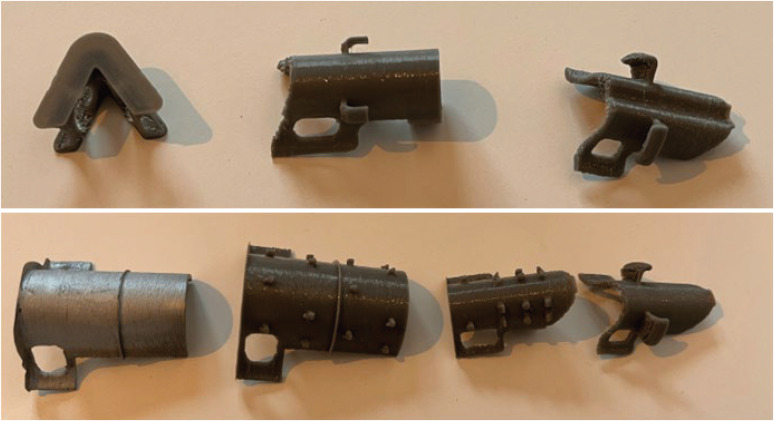
Overview of the main design iterations leading to the final EoloStent prototype.

Based on these objectives, the stent was developed through a systematic and iterative design process in which successive prototypes were generated, anatomically evaluated, and refined (Fig. 1). Initial design concepts aimed to increase airway patency by expanding the rima glottidis using a V-shaped supporting geometry. However, early fitting trials indicated that this configuration could interfere with complete epiglottic closure during simulated swallowing. Consequently, subsequent iterations redirected the expansion strategy toward the ventral portion of the rima glottidis, with the explicit aim of preserving dorsal structures involved in epiglottic sealing.

From the early stages, an open U-shaped cranial configuration was adopted to avoid full circumferential coverage of the laryngeal lumen and to reduce the risk of secretion retention. During preliminary fitting tests, prosthesis migration and rotational instability were identified as relevant failure modes. To improve cranial anchoring and positional stability, the cranial segment was opened and anatomically contoured to engage the cuneiform processes of the arytenoid cartilages. Small perforations were incorporated into this segment to facilitate optional suture-based fixation when required.

Caudally, dorsal displacement was observed during traction-based stability testing in early prototypes. To address this issue, a caudal fixation ring was incorporated to act as an anatomical stop and to improve retention within the laryngeal-tracheal transition. Alternative anti-migration strategies, including external protuberances similar to those used in Dumon-type stents, were initially considered but ultimately discarded due to concerns regarding focal mucosal trauma and pressure-related tissue injury.

The geometry and technical drawings were created using Rhinoceros 3D modeling software (Rhino 7 SR37; Robert McNeel & Associates, USA), and the final prototypes were manufactured using an HP MultiJet Fusion 3D (HP Jet Fusion 4200; HP, USA) printer with thermoplastic polyurethane (TPU) powder as the printing material. The CAD design process combined solid-based modeling with subsequent mesh-based refinement. Initial prototypes were developed using solid modeling techniques based on reference measurements of the glottic opening obtained from anatomical observations and endotracheal tube sizing. In later stages, design refinement was performed through iterative modifications of the 3D mesh models to improve anatomical conformity across different canine laryngeal morphotypes. These adjustments included modifications of component dimensions and angulations, particularly in the posterior arch and cranial anchoring arms, to optimize fixation, airway patency, and interaction with surrounding anatomical structures. Anatomical reference data were partially informed by CT measurements and direct morphometric observations, although the final device geometry was not individualized to a single specimen but instead optimized to accommodate common anatomical variations through size-based design.

Multiple design iterations were produced during development. Early prototypes included features such as external hooks and internal cavities intended to improve fixation or airflow; however, these were subsequently modified or removed based on functional testing. Design refinement was guided primarily by clinical-functional criteria, including preservation of epiglottic closure, resistance to migration, and adequate airway expansion while minimizing excessive radial force.

The TPU used had a Shore A hardness between 70 and 90, with wall thicknesses ranging from 0.5 to 1 mm, allowing sufficient flexibility for compression while maintaining structural stability. Thermoplastic polyurethane was selected for this prototyping phase to facilitate rapid design adjustments and anatomical fitting. For clinical application, however, future devices will be manufactured using medical-grade silicone, given its well-documented biocompatibility in airway stenting.

The device has been patented under the name EoloStent, in collaboration between the University of Zaragoza and the company EpiicVet (Epic Innova S.L., Spain). Five prosthesis sizes (XS to XL) were developed to accommodate the wide anatomical variability of the canine larynx. Prosthesis sizing was guided by endotracheal tube selection, with size equivalence and dimensional specifications summarized in Supplementary Fig. 1.

**Fig. 2:**
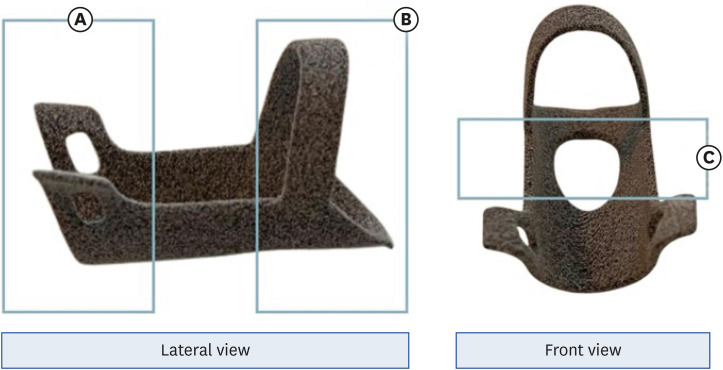
Lateral and front view of the laryngeal stent. (A) Cranial portion, (B) caudal body, and (C) central portion.

As illustrated in Fig. 2, the final prosthesis design consists of three anatomically contoured sections. The cranial portion features flexible arms designed to engage the cuneiform processes of the arytenoid cartilages, incorporating small perforations to allow suture-based fixation. The central body of the prosthesis is more rigid and semicircular in shape, providing structural support and enhanced ventral opening. This segment includes an oval orifice serving both as a safety feature and to facilitate mucociliary clearance. The caudal portion is equipped with an angled fixation ring, engineered to prevent distal migration into the digestive tract during swallowing and to secure the prosthesis within the upper trachea.

### Anatomical evaluation in canine cadavers

Eight canine cadavers of various breeds were used to evaluate the anatomical fit of the prosthesis and to identify potential complications related to placement and stability. All cadavers were obtained from client-owned animals that had been euthanized for reasons unrelated to the study, with prior informed consent from the owners for research use. Morphometric assessment focused on measuring the rima glottidis opening under three distinct conditions. Measurements were obtained via laryngoscopic imaging, using a metal rod with millimeter markings as a calibrated metric reference.

The width of the rima glottidis was selected as a key variable due to its relevance in preserving epiglottic function. An excessively widened glottic opening may compromise the ability of the epiglottis to close during swallowing, a phenomenon observed in arytenoid lateralization procedures and considered clinically significant.

**Fig. 3:**
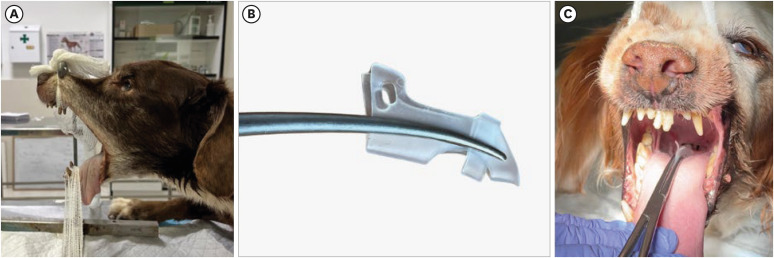
Implantation procedure. (A) Positioning of the cadavers for performing laryngeal measurements. (B) Compression of the device. (C) Direct visualization while introducing the stent.

Cadavers were positioned in standardized sternal recumbency, with the upper jaw suspended on a custom support and the lower jaw fixed to the surgical table, as shown in Fig. 3A. All measurements were performed in this consistent position.

To implant the stent, the device was manually compressed using long atraumatic forceps, inserting the caudal ring through the ventral opening of the larynx, as shown in Fig. 3B. While keeping the stent compressed, it was advanced through the glottis until fully positioned, with the cranial arms resting over the cuneiform processes 3C. The stent was then deployed by carefully opening the forceps and withdrawing them to allow controlled expansion in situ.

**Fig. 4:**
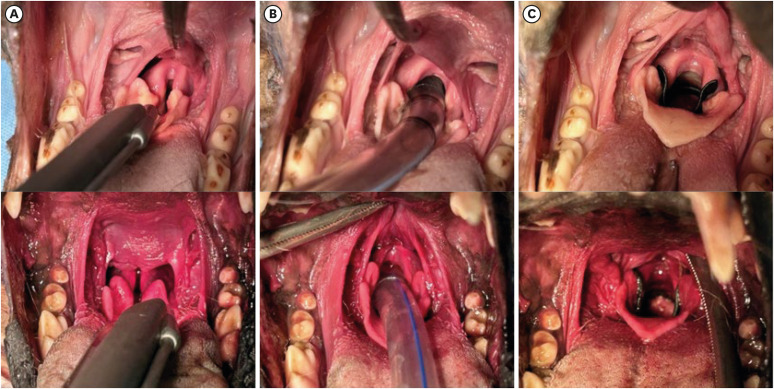
Visualization of the laryngeal opening. (A) Baseline (resting state), (B) after endotracheal intubation, and (C) after prosthesis implantation.

Rima glottidis (RG) dimensions were recorded under the following conditions (Fig. 4):

1. Baseline (resting state): without manipulation, using gentle traction of the tongue. This served as a reference measurement.
2. After endotracheal intubation (A-ET): the glottic opening was measured following placement of an appropriately sized endotracheal tube, always selected by the same veterinary surgeon.
3. After stent implantation (A-S): based on the diameter of the selected endotracheal tube, the corresponding prosthesis size was determined using a reference chart, and the rima glottidis was remeasured post-implantation.

To assess fixation stability, the prosthesis was subjected to controlled cranial and caudal traction applied at the central ventral orifice. The degree of displacement was classified as: absent (no displacement occurred without compression of the device), mild (slight movement was observed without dislodgement of the cranial arms or caudal ring) and, complete (the prosthesis could be displaced or removed entirely through traction alone). Finally, the integrity of the epiglottic seal was evaluated. Using forceps, the epiglottis was elevated to determine whether it achieved complete closure of the laryngeal inlet. This was recorded as a binary outcome (yes/no).

### Data analysis

Statistical analysis was performed using paired Student’s *t*-tests to compare glottic opening measurements before and after stent placement. Categorical variables, such as stent adaptation and displacement, were analyzed using Fisher’s exact test.

Given the very small sample size of several subgroups (n = 1–2), this study must be considered primarily descriptive. Accordingly, statistical tests have very limited power, and any reported *p* values should be interpreted as exploratory only.

Statistical analysis was performed with IBM SPSS 30.0 for Windows (IBM, USA), and error alpha was set at 0.050.

## RESULTS

A total of eight canine cadavers were included in this study. Of these, 50% were classified as mesocephalic (M) (4/8), 25% as dolichocephalic (D) (2/8), and the remaining 25% as brachycephalic (B) (2/8). Most animals were large breeds (4/8), with the Bracco being the most represented (2/8) (Table 1). Due to the lack of clinical history, the age of the specimens could not be determined.

**Table 1:** Individual specimen data

Breed	Morphotype	Baseline RG (mm)	A-ET RG (mm)	A-S RG (mm)	ET size (mm)	
					No. of stent	Stent size^a^
Mixed	M	4.3	14.6	11.1	7.0	M
Bracco	M	15.3	28.4	18.9	9.5	L^b^
Galgo	D	6.1	18.9	15.7	10.0	L
French Bulldog	B	2.8	10.2	11.4	5.5	S^b^
Yorkshire Terrier	M	4.0	14.2	10.3	6.0	S
German Shepherd	D	18.7	36.3	24.8	11.0	XL^b^
Bracco	M	7.3	10.1	12.2	10.0	XL
Chihuahua	B	0.8	2.3	2.2	5.0	XS

RG, Rima glottidis; A-ET, after endotracheal intubation; A-S, after stent implantation; ET, endotracheal tube; M, mesocephalic; D, dolichocephalic; B, brachycephalic.

^a^ET size refers to the internal diameter of the ET.

^b^Cases with suggested: non-optimal adaptation (undersized prosthesis).

The average RG at baseline was 7.72 ± 5.72 mm in mesocephalic dogs, 12.4 ± 9.91 mm in dolichocephalic, and 1.8 ± 1.41 mm in brachycephalic dogs. It is noteworthy that both brachycephalic animals showed evident laryngeal alterations. Despite morphological differences, no statistically significant differences were detected between groups (*p* = 0.153). Although no cadaver received the same endotracheal tube (ET) size, identical stent sizes were used in some animals based on stent equivalency chart. In total, one prosthesis of size XS and M were used, and two each of sizes S, L, and XL.

Laryngeal opening was analyzed after intubation (RG-TE) and after stent implantation (RG Stent), grouped by stent size. In the L group, the mean RG was 23.65 ± 6.72 mm with the ET and 17.30 ± 2.26 mm with the stent, a non-significant mean difference of 6.35 mm (*p* = 0.293) In the S group, the difference was 1.35 mm (12.20 vs. 10.85 mm), also not significant (*p* = 0.69). In the XL group (n = 2), the mean RG was 23.20 ± 18.53 mm with the ET and 18.52 ± 8.94 mm with the stent (*p* = 0.6155). For groups M and XS (n = 1 each), statistical analysis was not feasible, but observed absolute differences were 3.5 and 0.1 mm, respectively.

Overall, no statistically significant differences were observed between the glottic opening achieved with intubation and with the prosthesis, regardless of stent size. However, in all cases, the ET produced greater glottic opening than the stent (Table 2).

**Table 2:** Glottic opening by stent size compared to ET

Stent size	No.	A-ET (mm)		A-S (mm)		*p* value^a^
		Mean	SD	Mean	SD	
XS	1	2.30	-	2.20	-	-
S	2	12.20	2.85	10.85	0.7	0.691
M	1	14.20	-	20.30	-	-
L	2	23.60	6.70	17.35	2.2	0.293
XL	2	23.20	18.50	18.52	8.9	0.615

ET, endotracheal tube; A-ET, after endotracheal intubation; A-S, after stent implantation; SD, standard deviation.

^a^*p* values from paired Student’s *t*-test.

Stent performance was assessed using a functional composite approach that considered controlled glottic opening, preservation of epiglottic coverage, and resistance to displacement under traction. The objective was not maximal airway dilation, but rather the achievement of sufficient airway patency while maintaining laryngeal protective function.

The relationship between stent adaptation and prosthesis displacement (“drag”) was also evaluated. Optimal adaptation was achieved in 62.5% of cases (5/8), while 37.5% (3/8) were classified as non-optimal, designated as “small” (stent undersized based on ET chart). Stent displacement occurred only in animals with non-optimal adaptation (2/3), and never in those with optimal fit. Although this difference did not reach statistical significance (*p* = 0.107), the data suggest a possible link between misfitting and device instability.

All instances of prosthesis movement were classified as mild and consisted of minimal visible displacement without disengagement of the cranial anchoring arms from the cuneiform processes or loss of caudal fixation. No prosthesis could be completely displaced or removed by traction alone. In 100% of the cases, the epiglottis was able to fully cover the glottic opening, indicating preserved sealing function.

## DISCUSSION

Laryngeal stenosis, particularly due to paralysis or collapse, is a common cause of upper airway obstruction in dogs, significantly affecting their quality of life [1,9,11]. Traditionally, these conditions have been treated using surgical techniques such as unilateral arytenoid lateralization, ventriculocordectomy, or permanent tracheostomy [8,9,13,14,15]. Among these, arytenoid lateralization is considered the gold standard but is not without complications [8]. Reported rates of aspiration pneumonia range from 18% to 31.8%, with clinical recurrence due to suture failure and overall morbidity exceeding 50% in some series [4,13]. Furthermore, the need for general anesthesia, technical complexity, and high anatomical variability between patients complicate its urgent or universal application.

Considering these limitations, laryngeal stents have emerged as a less invasive and more immediately applicable alternative [2,17,19]. However, most existing models are adapted from human devices, such as Montgomery tubes or self-expanding metallic stents, whose efficacy in veterinary settings is limited by significant anatomical, histological, and functional differences between species. These cylindrical, closed-loop stents apply uniform radial pressure that can provide rapid laryngeal opening but may also cause complications such as migration, mucosal ulceration, granulation tissue formation, or interference with epiglottic closure [2,20,21].

The stent developed in this study was specifically designed to fit canine anatomy, featuring an open U-shaped cranial portion, a solid semicircular ventral body for structural support, and an angled caudal anchoring system to prevent migration. This configuration contrasts with traditional circular designs that mimic endotracheal tube-induced dilation and provide instantaneous airflow improvement [2,3,22]. However, such forced dilation can excessively open the rima glottidis and disrupt the physiology of swallowing, potentially increasing the risk of aspiration [7]. The iterative design process enabled by 3D printing was particularly relevant in this context, as minor variations in geometry had a clear impact on anatomical adaptation and prosthesis stability. The ability to refine the device design through successive prototypes was therefore essential to achieving an implant that balanced airway patency with preservation of epiglottic function.

Functionally, our results demonstrate that the glottic opening achieved with our prosthesis was consistently smaller than that obtained with endotracheal intubation. This controlled ventral dilation may be advantageous, as excessive widening of the rima glottidis, often associated with circular stents, can impair epiglottic closure and increase the risk of aspiration [4].

In contrast to conventional cylindrical devices, the prosthesis in this study was specifically designed to expand only the ventral portion of the glottis, preserving epiglottic sealing and minimizing dorsal pressure. This anatomical orientation, combined with the absence of displacement or over-dilation in properly sized implants, suggests that the radial forces exerted are limited, potentially reducing the risk of mucosal injury.

Furthermore, by avoiding full circumferential expansion, our design may also help prevent mucus stagnation. This concept is supported by recent evidence in human airway stenting, where fenestrated silicone stents demonstrated significantly lower rates of secretion retention (42.9% vs. 60.0%) and longer patency compared to conventional cylindrical stents. Computational simulations from that study further showed that fenestrations promote increased wall turbulence and preserve mucosal exposure, improving secretion clearance [18]. While such dynamics cannot be directly assessed in our cadaveric model, these findings reinforce the rationale for anatomically adapted, ventrally open stents in canine laryngeal applications.

As this was a cadaveric study, we were unable to assess tissue inflammation or nociception. However, similar laryngeal stents made of silicone have been used clinically in dogs with laryngeal paralysis, showing good tolerance and acceptable rates of complications, including mild inflammation or migration in some cases [2,3]. Notably, neither of these clinical reports conducted systematic histopathological evaluation of the tissue response.

In terms of stability, optimal stent adaptation was achieved in 62.5% of the specimens. Partial or suboptimal fitting led to mild displacement in two cases. One particularly illustrative case involved a French Bulldog, where the prosthesis was clearly undersized relative to the laryngeal anatomy. This discrepancy likely stemmed from the breed’s typical tracheal hypoplasia, requiring a smaller ET that underestimated the actual glottic diameter. Since the stent size was selected based on the ET used, the final implant was too small. This finding highlights the limitation of ET-based stent sizing, especially in brachycephalic breeds [11,13], and supports the use of larger prostheses or direct laryngeal measurements in such cases. Similarly, one cadaver required an ET size 11, which corresponded to the upper limit of the available stent range. Given the laryngeal dimensions observed, a larger stent would have been necessary to achieve optimal dilation, but such a size was not available. This highlights the need to extend the range of manufactured stent sizes for clinical application.

Compared to other devices in the literature, such as the solid stent described by Bourinet et al. [23] or the V-shaped stents by Cabano et al. [19], our prosthesis offers functional opening while preserving the anatomical contours of the rima glottidis and allowing for epiglottic closure. Previous models have shown improved respiratory flow but also increased resistance with the epiglottis closed and a higher risk of migration or malposition, especially with self-expanding metallic designs [19,23].

Biomechanically, implant stability must also be considered in the context of laryngeal rigidity distribution. The canine larynx presents a distinct vertical stiffness gradient, with greater mechanical resistance in the caudal and dorsal segments of the glottis [5]. Compared to the human larynx, dogs have a thicker superficial layer and a less defined ligamentous structure, which affects their elastic response to expansion forces [10]. In this context, circular stents may apply excessive radial force as endotracheal tubes, that is unevenly distributed, increasing the risk of localized compression and migration [2,20].

The open design of the prosthesis evaluated here, with targeted anchoring arms and a semicircular body, aims to respect the larynx’s natural biomechanics by providing support to the more stable regions while avoiding excessive pressure on mobile or sensitive zones. This anatomical and mechanical alignment may explain why, despite producing smaller glottic openings than an ET, the implant remained stable and less likely to interfere with epiglottic closure or swallowing.

It is important to note that this study was conducted on cadavers, limiting the ability to evaluate inflammatory responses, long-term tolerance, or clinical efficacy during swallowing and phonation. Moreover, due to the small sample size, and particularly the very small number of animals within several subgroups, the study must be considered primarily descriptive and statistically underpowered. As a consequence, the reported *p* values are exploratory and should not be interpreted as evidence of true equivalence or absence of effect. Nonetheless, the cadaveric model allowed for an objective assessment of anatomical fit and implant mechanics, which are critical in the early development stages of medical devices. Future studies should include in vivo trials to assess the prosthesis’s function and stability under real respiratory cycles, as well as its interaction with mucosal secretions, tissue response, and long-term tolerability.

## SUPPLEMENTARY MATERIAL

Supplementary Fig. 1 Dimensional specifications and recommended endotracheal tube equivalence for the five EoloStent sizes designed for canine laryngeal applications. Courtesy of Epiicvet (Epic Innova S.L., Spain).
